# The Association between Omega-3 Fatty Acid Intake and Human Brain Connectivity in Middle-Aged Depressed Women

**DOI:** 10.3390/nu12082191

**Published:** 2020-07-23

**Authors:** Seon-Joo Park, Do-Kyung Lee, Bokyoung Kim, Kyoung-Sae Na, Chang-Ho Lee, Young-Don Son, Hae-Jeung Lee

**Affiliations:** 1 Department of Food and Nutrition, College of BioNano Technology, Gachon University, Gyeonggi 13120, Korea; chris0825@hanmail.net; 2 Department of Health Sciences and Technology, GAIHST, Gachon University, Incheon 21999, Korea; boknyu88@naver.com; 3 Department of Biomedical Engineering, College of Health Science, Gachon University, Incheon 21936, Korea; kbk86133032@gmail.com; 4 Department of Psychiatry, Gachon University Gil Medical Center, Incheon 21565, Korea; ksna13@gmail.com; 5 Research Group of Functional Food Materials, Korea Food Research Institute, Wanju 55365, Korea; chang@kfri.re.kr

**Keywords:** depression, omega-3 fatty acid, resting-state functional MRI, middle-aged women

## Abstract

Omega-3 fatty acid (*n*-3 FA) intake is known to have a preventive effect on depressive symptoms in a general population. This study assessed the effects of *n*-3 FA intake on depressive symptoms and brain function in middle-aged women. Depressive symptoms were screened using the Beck Depression Inventory-II (BDI-II) and Center for Epidemiologic Studies-Depression scale (CES-D) assessment questionnaires, and *n*-3 FA intakes were assessed using semiquantitative food frequency questionnaire. We found that *n*-3 FA intakes were negatively associated with depressive symptoms in middle-aged women. Psychiatrists diagnosed the presence of depressive disorders using the 5th edition of the Mental Disorder Diagnosis and Statistics Manual (DSM-5). Resting-state functional magnetic resonance imaging (rs-fMRI) was performed to investigate the association between *n*-3 FA intake and brain functional connectivity. Functional connectivity of the right middle frontal cortex (default mode network) and the right middle temporal pole (frontoparietal network) was positively associated with depressive symptom scores and negatively associated with *n*-3 FA intakes. In conclusion, high *n*-3 FA intake decreases the risk of depressive symptoms and modifies the brain functional connectivity in middle-aged women.

## 1. Introduction

Depressive disorder is one of the most prevalent psychiatric disorders in the world. The World Health Organization has estimated that more than 264 million people have depressive symptoms, and more women are affected by depression than men worldwide [1]. In Korea, 6.7% (male 4.2%, female 9.1%) of adults aged >19 years had depression, based on responses to Patient Health Questionnaire (PHQ)-9, with adults aged ≥70 years having the highest prevalence of depression (11.2%) [2]. Menopause is associated with depression in women because estrogen deficiency may increase susceptibility to depression [3]. A meta-analysis revealed that later onset of menopause was associated with a reduced risk of severe depression [4]. In a longitudinal observational study, peri-menopausal and postmenopausal women were at a higher risk of depression than premenopausal women [5].

Among nutritional factors, polyunsaturated fatty acids (PUFAs) are considered to have potential protective components against depressive disorders [6,7]. A meta-analysis suggested a beneficial effect of omega-3 PUFA supplementation on major depressive disorder (MDD) [8]. In addition, a review paper reported that omega-3 PUFA, especially eicosapentaenoic acid (EPA), may be more beneficial than docosahexaenoic acid (DHA) in mood disorder treatment [9]. A two-month, randomized, double-blind, placebo-controlled trial revealed that omega-3 PUFA supplementation reduced the occurrence of depressive symptoms in elderly females [10]. Moreover, a longitudinal cohort study showed that high intakes of EPA and DHA reduced the risk of depression in middle-aged Japanese men and women [11].

Following recent advancements in neuroimaging technology, depressive disorders have been studied from structural and functional perspectives [12]. Functional magnetic resonance imaging (fMRI) has revealed altered brain connectivity in specific resting-state neural networks in people with depression [13]. It has also been reported that an imbalance in brain connectivity may be involved in the pathophysiology of MDD [14].

Some studies have shown the effects of omega-3 fatty acids in the brain. A review paper reported that omega-3 fatty acid supplementation could protect against neurodegeneration in older adults [15]. Moreover, other studies have reported that EPA and DHA supplements can reduce or increase functional brain activation [16,17,18]. However, it remains unexplored as to which mechanism omega-3 fatty acids use to alter brain connectivity [19].

This study aimed to examine the association between omega-3 fatty acid and depression in middle-aged women and to identify the brain connectivity associated with fatty acid intake and score of depression scales (Beck Depression Inventory-II (BDI-II) and Center for Epidemiologic Studies-Depression scale (CES-D)) among depressed subjects by using resting-state (rs)-fMRI-based analysis.

## 2. Materials and Methods

### 2.1. Subjects

This diet-depression cohort study was conducted to identify dietary and environmental factors related to depression in Korean middle-aged women. The participants in the first wave of the cohort study included 2200 females aged 45–69 years. They were recruited through hospital and community health centers in the Seoul and Gyeonggi areas in South Korea and voluntarily participated in the study. The first wave of examination began in October 2016 and ended in November 2018. 

The study was approved by the Institutional Review Board of the Gachon University Gil Medical Center (GDIRB2016-271) and was conducted in accordance with the Declaration of Helsinki. All subjects gave their informed consent for inclusion before they participated in the study, and all surveys were conducted through face to face interviews.

### 2.2. Methods

#### 2.2.1. Depressive Symptom Assessment

Depressive symptoms were assessed using the BDI-II and CES-D. The BDI-II contains 21 questions, with each answer scored on a scale of 0 to 3 and the total score range is from 0 to 63. A higher total score indicates more severe depressive symptoms [20]. The Korean version of BDI-II is considered as a validated tool to assess depressive symptoms in patient and normal subject populations [21]. We classified people with a BDI-II score of 14 or higher as people with depressive symptoms. The CES-D questionnaire comprises 20 questions and the total score range is from 0 to 60, with lower scores indicating fewer depressive symptoms. The scale is reported to be a valid and reliable metric, and a cut-off score of ≥16 is used to identify subjects with relevant depressive symptoms [22]. 

#### 2.2.2. Nutritional Assessment

Dietary intakes, including mean nutrient intake per day and omega-3 fatty acid intake per day, were assessed using the previously validated 108-item Semiquantitative Food Frequency Questionnaire (SQ-FFQ) [23,24]. The frequency of food intake was assessed over 9 categories (almost never, 1 time/month, 2–3 times/month, 1 time/week, 2–4 times/week, 5–6 times/week, 1 time/day, 2 times/day, and 3 times/day). Serving size was assessed as small (0.5 serving), medium (1 serving), or large (1.5 serving). Nutrient intakes were calculated using the food composition database created by the Rural Development Administration of Korea [25]. A fatty acid database developed in a previous study was also used [26].

#### 2.2.3. Other Variables

Education level was categorized into four groups: elementary school graduation or less, middle school graduation, high school graduation, and college graduation or higher. Household income was categorized into four groups: <1,000,000 won, 1,000,000–2,000,000 won, 2,000,000–4,000,000 won, >4,000,000 won. Current smoking status was classified as current smoker or non-smoker. Alcohol drinking status was classified as current drinker or non/ex-drinker. Marital status was classified as married or other (widowed, single, and others). Job type was categorized as white-collar worker, service worker, blue-collar worker, or housewife. Chronic disease status was determined by the response to the following question. “Have you ever been diagnosed with diabetes, hypertension, heart disease, or cancer by a physician?” Responses were classified as yes or no. Physical activity question (“Do you exercise regularly enough to breathe and sweat?”) responses were classified as yes or no. Menopausal status was classified as yes or no.

Height and weight were measured to the nearest 0.1 cm and 0.1 kg, respectively, by trained staff using a scale and a wall-mounted extensometer. Body mass index (BMI) was calculated as the weight in kilograms/height in meters squared.

#### 2.2.4. Statistical Analysis

The characteristics of the study subjects are expressed as a percentage (categorical variables) or as mean and standard deviation values (continuous variables). Continuous variables were used to compare cases and controls by using independent *t*-tests, and chi-squared tests were used for categorical variables. Odds ratios (OR) and 95% confidence intervals (CI) were computed for the association between omega-3 fatty acid intake and depressive symptoms using multivariable logistic regression analysis with adjustments for age, BMI, education level, household income, marital status, job, current alcohol drinking, current smoking, physical activity, chronic diseases status, sleep duration, family history of depression, stress, menopausal status, and total energy intake. Omega-3 fatty acid intake was categorized into quartiles, with the lowest quartile considered the reference level.

Subjects with implausible energy intake values (<500 kcal/day and >3500 kcal/day) were excluded from the analyses [27]. From the 2200 subjects in the cohort, we analyzed data for 2190 participants. All statistical analyses were performed using SAS software (version 9.4 SAS institute Inc., Cary, NC, USA) and statistical significance was accepted at *p* < 0.05.

#### 2.2.5. Scan Protocol for Resting-State fMRI

Among the 2200 subjects in the study cohort, 130 subjects (45 normal subjects and 85 depressive symptom subjects) were selected to undergo resting-state fMRI (rs-fMRI). Depressive symptom subjects were selected from those who had a BDI-II score of 14 or more and a CES-D score of 16 or higher. The selected normal subjects had a sum of BDI-II and CES-D scores of less than 10. Before rs-fMRI scanning, the presence of depressive disorders of 130 subjects was diagnosed by a psychiatrist using the Diagnostic and Statistical Manual of Mental Disorders, Fifth Edition (DSM-5) [28].

For scanning, the subjects were to lie down comfortably on the patient bed of the MRI system. They were instructed to open their eyes, stare at a dot on the screen, and stay awake during scanning. Subject head position was fixed tightly using sponges, and earplugs were worn to minimize machine noise.

For each subject, two sessions of rs-fMRI were acquired, and each session comprised a time series of 125 volumes. Gradient-recalled echo-planar imaging sequences were obtained using the following parameters; matrix = 64 × 64, voxel size = (3.75 × 3.75 × 4.0) mm, number of slices = 38, TR/TE = 2500/30 ms, NEX = 1, FA = 9°, and BW = 2170 Hz. For structural imaging, T1-weighted image scans were acquired by performing three-dimensional magnetization-prepared rapid acquisition with gradient echo (3D-MPRAGE) with the following parameters; matrix = 448 × 512, voxel size = (0.5 × 0.5 × 1.0) mm, number of slices = 176, TR/TE = 1900/3.03 ms, T1 = 900 ms, NEX = 1, FA = 9°, and BW = 160 Hz.

#### 2.2.6. First-Level Analysis of Functional Connectivity

The rs-fMRI images were preprocessed using SPM12 (Statistical Parametric Mapping, Wellcome Trust Centre for Neuroimaging, London, UK). First, the rs-fMRI images were realigned to the first image for motion correction and an average image of the realigned images was obtained. The T1 images were segmented with three compartments (gray matter, white matter, and cerebrospinal fluid). Skull-strip processing was performed by combining these three image segments. Next, the skull-stripped T1 image was co-registered to the averaged rs-fMRI image. The co-registered T1 images were normalized to the Montreal Neurological Institute (MNI) standard template. Using the registration parameters, the corresponding rs-fMRI images were resampled to the MNI template using (3 × 3 × 3) mm voxel sizes. The normalized rs-fMRI images were smoothed with an 8 mm full width at half maximum three-dimensional Gaussian kernel.

Independent component analysis (ICA) was used to decompose the blood oxygen level-dependent images of the whole brain into independent network signals by using the functional connectivity toolbox (CONN18a). Group ICA was performed with a specific number of independent components (ICs) and was optimized by changing the number of ICs from 10 to 30. For each subject, beta-maps of each deconvolved ICs were obtained. During ICA, five well-known networks—the default mode network (DMN), salience network (SN), left and right frontoparietal networks (FPN), and the dorsal attention network (DAN)—were identified automatically by selecting the beta-maps with the highest average values within a specific region of interest (ROI) as predefined in CONN18a.

#### 2.2.7. Second- and Third-Level Analysis of Functional Connectivity

To find brain regions differentially affected by depression severity, beta-maps were analyzed in subjects from the depressive symptoms group (*n* = 76). For second-level analysis, linear regression analysis was performed using SPM12 for beta-maps of the major network with depression-related CES-D and BDI-II scores. The inference threshold for statistical significance was set to *p* < 0.001 (uncorrected) and cluster size was set to 20 voxels. The ROIs of each cluster (ROI_cluster_) were generated by assessing inference thresholds from the resultant t-map of the regression analysis. The averaged values of the beta-map of the decomposed IC within the ROI_cluster_ were obtained.

As a third-level analysis, in order to investigate the correlation between the depression severity-related ROI_cluster_ and the omega-3 fatty acid intake, the Pearson’s correlation coefficient between the beta-values within the ROI_cluster_ and the omega-3 fatty acid intake was calculated by using the Statistical Package for the Social Sciences (SPSS Statistics 25, IBM). Age was considered as a covariate. Statistical significance of the obtained correlation was set at *p* < 0.05.

## 3. Results

### 3.1. Characteristics of the Study Participants

Among the 2190 participants, 487 subjects (22.2%) were identified as having depressive symptoms (BDI-II score ≥ 14). The characteristics of the cases (depressive symptom subjects) and controls (normal subjects) are summarized in Table 1. Compared with normal subjects, participants who presented with depressive symptoms had a lower household income, less physical activity, higher smoking, had family history of depression, less sleeping time, had higher levels of stress, and fewer were married. However, there were no significant differences between the two groups in age, BMI, education level, current alcohol drinking, job, menopausal status, and chronic disease status.

### 3.2. Analysis of Nutrient Intakes of Normal and Depressive Symptoms Groups

Table 2 shows that the intake of most nutrients—PUFA, omega-3 fatty acid, omega-6 fatty acid, alpha-linolenic acid (α-LA, 18:3 *n*-3), eicosapentaenoic acid (EPA, 20:5 *n*-3), docosapentaenoic acid (DPA, 22:5 *n*-3), and docosahexaenoic acid (DHA, 22:6 *n*-3)—was significantly lower in depressive subjects than in normal subjects except for energy and carbohydrate intakes.

### 3.3. Association between Omega-3 Fatty Acid Intake and Depressive Symptoms

Multivariable-adjusted regression analysis showed that the risk of depressive symptoms was negatively associated with omega-3 fatty acids, especially EPA, DPA, and DHA, after adjusting for age, BMI, physical activity, current smoking, current alcohol drinking, marital status, education, household income, job, sleep duration, chronic diseases status, family history of depression, stress, and total energy intake.

Adjusted OR and 95% CI of depressive symptoms related to omega-3 fatty acids intake level are summarized in Table 3. In model 1, PUFA, total omega-3 fatty acid, α-LA, EPA, DPA, and DHA showed significant linear relationship trends. In model 2, compared with subjects in the lowest quartile of the omega-3 fatty acids intake, those in the highest quartile had a significantly lower odds of depressive symptoms (OR = 0.63, 95% CI: 0.42–0.96, *p* for trend = 0.04 for total omega-3 fatty acid; OR = 0.61, 95% CI: 0.43–0.89, *p* for trend = 0.0186 for EPA; OR = 0.54, 95% CI: 0.38–0.78, *p* for trend = 0.0012 for DPA; OR = 0.61, 95% CI: 0.42–0.88, *p* for trend = 0.011 for DHA).

### 3.4. Functional Connectivity of the Within-Group Analysis

**Figure 1:**
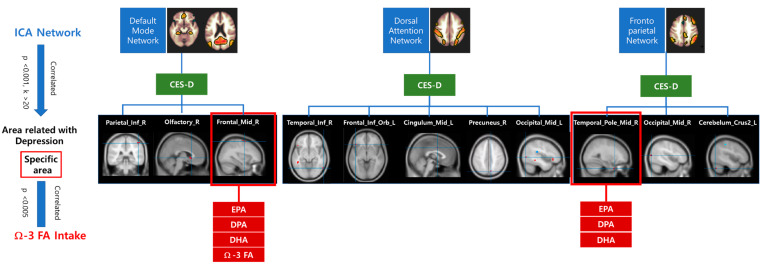
The beta-maps of the default mode network (DMN), dorsal attention network, and frontoparietal network were related with CES-D score. CES-D: Center for Epidemiological Studies-Depression Scale, Parietal_Inf_R: right inferior parietal, Olfactory_R: right olfactory, Frontal_Mid_R: right middle frontal cortices, Temproral_Inf_R: right inferior temporal, Frontal_Inf_Orb_L: left inferior orbital frontal, Cingulum_Mid_L: left middle cingulum, Precuneus_R: right precuneus, Occipital_Mid_L: left middle occipital cortices, Temporal_Pole_Mid_R: right middle temporal pole, Occipital_Mid_R: right middle occipital cortex, Cerebellum_Crus II_L: left cerebellar cortical crus II.

In the DMN, as the CES-D scores increased, the functional connectivity of the right inferior parietal, right olfactory, and right middle frontal cortices increased. In the DAN, CES-D scores were positively associated with the functional connectivity of the right inferior temporal, left inferior orbitofrontal, left middle cingulate, right precuneus, and left middle occipital cortices. In the FPN, as the CES-D score increased, involvement of the right middle temporal pole, right middle occipital cortex, and left cerebellar cortical crus II significantly increased (Figure 1).

Among the assessed brain regions, involvement of the right middle frontal cortex of the DMN showed a significant negative correlation with the intake of omega-3 fatty acid (*r* = −0.32, *p* = 0.005), EPA (*r* = −0.35, *p* = 0.002), DPA (*r* = −0.35, *p* = 0.002), and DHA (*r* = −0.35, *p* = 0.002), and the right middle temporal pole of the frontoparietal network also had a significant negative correlation with the intake of EPA (*r* = −0.36, *p* = 0.002), DPA (*r* = −0.38, *p* = 0.001), and DHA (*r* = −0.36, *p* = 0.001) (Table 4).

## 4. Discussion

An accumulation of epidemiological research results has revealed that a high intake of omega-3 fatty acids (α-LA, EPA, and DHA) can decrease the risk of MDD [9,29]. The effectiveness of EPA and DHA in the treatment of depressive symptoms in unipolar and bipolar depression was reported in a review paper [30]. This study also showed the inverse associations between omega-3 fatty acid intake levels (EPA, DPA, and DHA) and depressive symptoms in Korean middle-aged women.

In a randomized controlled trial study, 2.5 g/day of *n*-3 long-chain PUFA significantly decreased Geriatric Depression Scale scores after 2 months of supplementation among depressed females aged 66–95 years [10]. In the Seguimiento Universidad de Navarra (SUN) cohort study, the highest quartile of the omega-3 PUFA intake group had a lower risk of mental disorder presence compared to the risk in the lowest intake group [31]. Moreover, it has been reported that erythrocyte DHA composition was significantly lower (−20%) in MDD subjects than in healthy controls [32] and that the plasma EPA level was inversely associated with the presence of depressive symptoms among the elderly in France [33]. In Korea, several small intervention studies have reported a negative association between omega-3 fatty acid intake and depression. However, these studies showed either a null or positive direction results [34,35,36]. A cross-sectional study reported that depressed women (*n* = 151) consumed less omega-3 fatty acids than non-depressed women (*n* = 641) among Korean women aged 50–64 years [36]. 

The pathophysiological mechanism of the effect of omega-3 fatty acid on depression may be related to the increase in the ratio of omega-6/omega-3 PUFAs, which could activate the secretion of proinflammatory cytokines, in turn activating the hypothalamic pituitary adrenal (HPA) axis, increasing cortisol production, and decreasing serotonin producer availability [37]. Moreover, omega-3 fatty acids such as DHA and EPA provide cell membrane fluidity and facilitate neurotransmission and ion channel flow processes, which have major roles in brain development and brain function [38]. In addition to fatty acids, fiber and phytonutrients in vegetables, fruits, and complex carbohydrates may alleviate depression through changes in gut microbiota which could affect neurotransmitter metabolism [39] 

In this study, we investigated the association between omega-3 fatty acid intake and brain functional connectivity using rs-fMRI. To the best of our knowledge, this is the first study to examine the associations between omega-3 fatty acid intake, depressive symptoms, and brain connectivity in middle-aged women in Korea. The right middle frontal cortex region of the DMN and the right middle temporal pole region of the FPN showed strong inverse associations (*p* < 0.005) with EPA, DPA, and DHA intake levels. Although they have not looked at the same region of the brain, previous studies have reported that intake of omega 3 fatty acids is associated with brain connectivity. In a randomized controlled trial, 2.2 g/day of fish oil (long chain omega-3 fatty acid) intake over 26 weeks significantly increased gray matter volume in the right middle temporal gyrus and improved executive functions, which were positively correlated with omega-3 fatty acid indices, in the treatment group compared to the placebo group (50–75-year-old males and females) [18]. Furthermore, following fish oil supplementation in adolescents with MDD, erythrocyte EPA level was positively correlated with choline level in the right dorsolateral prefrontal cortex (DLPFC), and erythrocyte DHA level was negatively correlated with myo-inositol concentration in the left DLPFC [40]. In addition, resting-state functional connectivity between task regions (precentral gyrus and middle frontal gyrus) and DMN regions (medial frontal gyrus and precuneus) decreased with supplementation of the mixture of omega-3 fatty acid, green tea catechins, and ginsenoside, compared to that from the placebo treatment, suggesting an increase in the segregation of task- and rest-related brain activities [41].

It has also been reported that an EPA-rich supplement can reduce functional activation in the left anterior cingulated cortex and increase functional activation in the right precentral gyrus, whereas a DHA-rich supplement can increase functional activation in the right precentral gyrus during spatial Stroop working memory tasks in young adults [16]. In our study, the left cingulum middle region of a DAN showed an inverse association with omega-3 fatty acid and α-LA intakes, and the right temporal inferior region of a DAN had an inverse association with EPA, DPA, and DHA intake. 

In other studies, the DHA composition of postmortem-assessed orbitofrontal cortices in MDD patients was significantly lower (−22%) than that in normal control subjects. A DHA deficiency in the orbitofrontal cortex, which has an important role in hedonic and emotional processes, may be considered a potential etiology of MDD [42,43]. In our study, the positive association between the involvement of the left frontal inferior orbital region and the CES-D score was also observed, but did not show any association with omega-3 fatty acid intake. 

The most consistent brain-related observation in major depression is decreased frontal lobe function (mainly involving the medial prefrontal cortex) and increased limbic system activity (amygdala) [44]. In depressed patients, there is decreased cortical regulation of limbic activation in response to negative stimulation [14]. A meta-analysis revealed that network dysfunction in MDD is related to resting-state functional connectivity: hypoconnectivity in the frontoparietal network seeds and regions of the bilateral posterior parietal cortex, which control attention and emotion regulation, and hyperconnectivity in the default network seeds and regions of the hippocampus (middle temporal gyrus and medial prefrontal cortex), which support self-referential thinking and effective decision-making [45]. Greicius et al. [13] reported that resting-state subgenual cingulate and thalamic connectivities were significantly greater in depressed subjects than in healthy control subjects. These regions could be involved in the reduced activation of the dorsal anterior cingulate cortex, which controls negative emotional response.

The FPN is reported to have a top-down regulatory function in attention and emotion [45]; in addition, the right inferior parietal lobe response during somatosensory stimulation was observed to be significantly higher in MDD subjects than in schizophrenic subjects [46]. In a postmortem study, elderly MDD patients had reduced right and left orbital frontal cortex volumes, suggesting that the orbital frontal cortex region may have a role in the development of depression [47].

This study has several limitations. First, it is a cross-sectional study, and the effect of omega-3 fatty acid on brain functional connectivity is unknown. Second, as we used self-administered instruments—BDI-II and CESD—the observed symptoms of depression could not be fully reflected in the scores of each instrument. If we added an interviewer-rated scale, such as the Hamilton Rating Scale for Depression (HRSD), we could have comprehensively assessed depressive symptoms by including both subjective and objective symptoms. Third, only subjects with selected depressive symptoms were included in the analysis of brain connectivity and omega-3 fatty acid intakes. However, a broader selection of subjects was not within our research purpose and scope. Regression analysis of the relationship of functional connectivity with depressive symptoms assessed by CES-D revealed altered functional connectivity was associated with depression severity; as a result, the brain regions were investigated to determine if their alterations were related to changes in omega-3 fatty acid intake. The negative correlation between depression-related brain regions and fatty acid intake may support results in previous studies showing that depressed patients have lower blood omega-3 fatty acid or DHA levels than normal subjects. More longitudinal studies with large sample sizes, including normal subjects, are needed to determine the comprehensive effects of omega-3 fatty acid intake on brain functional connectivity.

There have been several published meta-analyses and systematic review papers about rs-fMRI and MDD [44,45,48]. Although the use of rs-fMRI for MDD assessment can provide information on the potential pathophysiology of depression, standardization of rs-fMRI study design and analytic strategies, as well as increasing sample size, are needed to more fully elucidate the mechanisms involved in MDD.

In conclusion, the functional connectivity of depression-related brain structures (right middle frontal of DMN and right middle temporal pole of FPN) were negatively associated with omega-3 fatty acid intake. The results suggest that these brain regions can be modified by the intake of omega-3 fatty acids, thereby protecting against depression development in Korean middle-aged women.

## Figures and Tables

**Table 1:** Baseline characteristics of normal and depressive symptom groups.

	Normal (*n* = 1703)	Depressive Symptoms (*n* = 487) *	*p*-Value
Age (years), mean ± SD	58.3 ± 5.8	57.8 ± 5.9	0.167
BMI (kg/m^2^), mean ± SD	24.2 ± 3.2	24.0 ± 3.4	0.268
BDI-II score, mean ± SD	5.6 ± 3.6	21.0 ± 7.0	<0.0001
CES-D score, mean ± SD	5.6 ± 5.2	18.7 ± 9.9	<0.0001
Education level, *n* (%)			0.18
Elementary school	237 (13.9)	85 (17.5)	
Middle school	448 (26.3)	121 (24.8)	
High school	751 (44.1)	216 (44.4)	
College and higher	267 (15.7)	65 (13.3)	
Household income, *n* (%)			<0.0001
<1,000,000 won	123 (7.2)	61 (12.5)	
1,000,000–2,000,000 won	324 (19.0)	126 (25,9)	
2,000,000–4,000,000 won	635 (37.3)	166 (34.1)	
4,000,000 won	621 (36.5)	134 (27.5)	
Current Smoking, *n* (%)			0.0001
No	1661 (97.5)	458 (94.1)	
Yes	42 (2.5)	29 (6.9)	
Current alcohol drinking, *n* (%)			0.932
No	1189 (69.8)	341 (70.0)	
Yes	514 (30.2)	146 (30.0)	
Physical activity, *n* (%)			<0.0001
No	662 (38.9)	245 (50.3)	
Yes	1041 (61.1)	242 (49.7)	
Marital status, *n* (%)			<0.01
Married	1340 (78.7)	349 (71.7)	
Others	363 (21.3)	138 (28.3)	
Job, *n* (%)			0.329
White-collar worker	141 (8.3)	28 (5.7)	
Service worker	383 (22.5)	114 (23.4)	
Blue-collar worker	158 (9.3)	47 (9.7)	
Housewife	1021 (60.0)	298 (61.2)	
Chronic disease, *n* (%)			0.269
No	1130 (66,3)	310 (63.7)	
Yes	573 (33.7)	177 (36.3)	
Family history of depression, *n* (%)			0.0005
No	1681 (98.7)	469 (96.3)	
Yes	22 (1.3)	18 (3.7)	
Sleep duration, *n* (%)			0.0387
<6 h	280 (16.5)	104 (21.4)	
6–8 h	1122 (65.9)	298 (61.2)	
>8 h	300 (17.6)	85 (17.5)	
Stress, *n* (%)			<0.0001
Rarely	514 (30.2)	35 (7.2)	
A litter	909 (53.4)	179 (36.8)	
A lot	269 (15.8)	251 (51.5)	
Very much	11 (0.7)	22 (4.5)	
Menopausal status			0.809
No	192 (11.3)	53 (10.9)	
Yes	1511 (88.7)	434 (89.1)	

* Depressive symptoms: BDI-II ≥ 14. SD: standard deviation, BDI-II: Beck Depression Inventory-II, CES-D: Center for Epidemiological Studies-Depression Scale.

**Table 2:** Baseline nutrient and fatty acid intakes in study groups.

	Normal (*n* = 1703)			Depressive Symptoms (*n* = 487)			*p*-Value
Energy (kcal)	1352.2	±	375.5	1315.8	±	372.7	0.059
Protein (g)	46.0	±	16.8	43.5	±	16.1	0.0003
Fat (g)	29.9	±	14.2	28.1	±	13.7	0.0097
Carbohydrate (g)	218.9	±	56.4	215.7	±	55.9	0.265
Fiber (g)	5.4	±	2.2	5.1	±	2.2	0.0007
Calcium (mg)	404.6	±	190.0	369.5	±	189.8	0.0003
Phosphorous (mg)	783.5	±	290.6	727.4	±	276.4	0.0001
Fe (mg)	10.8	±	4.2	10.3	±	4.4	0.02
Sodium (mg)	2926.4	±	1371.8	2788.2	±	1393.2	0.051
K (mg)	2122.5	±	794.7	1968.4	±	778.2	0.0002
Vitamin A (RE)	514.6	±	271.2	468.0	±	268.4	0.0008
Carotene (µg)	2488.8	±	1412.0	2279.5	±	1420.4	0.004
Retinol (µg)	86.4	±	64.7	75.1	±	57.8	0.0002
Vitamin B_1_ (mg)	1.3	±	0.4	1.2	±	0.4	0.003
Vitamin B_2_ (mg)	0.9	±	0.4	0.9	±	0.4	0.012
Niacin (mg)	9.8	±	3.9	9.3	±	3.9	0.023
Vitamin C (mg)	64.5	±	37.8	56.8	±	35.2	<0.0001
Polyunsaturated fatty acid (mg)	8.0	±	4.0	7.4	±	3.9	0.0028
Omega-3 fatty acid (mg)	0.9	±	0.6	0.8	±	0.5	<0.0001
Omega-6 fatty acid (mg)	7.1	±	3.5	6.6	±	3.4	0.0041
Alpha-linolenic acid *	562.5	±	308.8	520.6	±	306.8	0.0083
Eicosapentaenoic acid *	109.7	±	100.2	91.4	±	80.4	<0.0001
Docosapentaenoic Acid *	15.4	±	16.2	12.1	±	12.0	<0.0001
Docosahexaenoic acid *	170.2	±	164.1	139.9	±	131.1	<0.0001

* mg per 100 g of total fatty acid.

**Table 3:** Association between omega-3 fatty acid intake and the risk of depressive symptoms obtained by multivariate-adjusted logistic regression analysis.

						Model 1				Model 2		
		Median	No. of Total	No. of Cases		Odds Ratio (95% CI)				Odds Ratio (95% CI)		
Polyunsaturated fatty acid (PUFA)	Q1	3.8	547	150	Q1	1.00			Q1	1.00		
	Q2	6.1	548	118	Q2	0.72	0.55	0.95	Q2	0.69	0.50	0.96
	Q3	8.4	548	113	Q3	0.68	0.52	0.90	Q3	0.66	0.46	0.94
	Q4	12.3	547	106	Q4	0.63	0.47	0.84	Q4	0.63	0.41	0.98
*p*-value for trend						0.0025				0.0585		
Total omega-3 fatty acid	Q1	0.4	547	146	Q1	1.00			Q1	1.00		
	Q2	0.6	548	129	Q2	0.84	0.64	1.10	Q2	0.78	0.57	1.07
	Q3	0.9	548	107	Q3	0.66	0.50	0.88	Q3	0.63	0.45	0.90
	Q4	1.4	547	105	Q4	0.65	0.49	0.86	Q4	0.63	0.42	0.96
*p*-value for trend						0.0019				0.04		
Alpha-linolenic acid (α-LA)	Q1	254.9	547	140	Q1	1.00			Q1	1.00		
	Q2	411.4	548	126	Q2	0.86	0.65	1.13	Q2	0.93	0.67	1.28
	Q3	578.4	548	113	Q3	0.74	0.56	0.99	Q3	0.76	0.54	1.08
	Q4	894.3	547	108	Q4	0.70	0.53	0.93	Q4	0.74	0.49	1.13
*p*-value for trend						0.0131				0.1237		
Eicosapentaenoic acid (EPA)	Q1	29.4	547	147	Q1	1.00			Q1	1.00		
	Q2	60.1	548	117	Q2	0.74	0.56	0.98	Q2	0.77	0.57	1.06
	Q3	102.2	548	129	Q3	0.84	0.64	1.10	Q3	0.80	0.58	1.11
	Q4	198.9	547	94	Q4	0.57	0.42	0.76	Q4	0.61	0.43	0.89
*p*-value for trend						0.0006				0.0186		
Docosapentaenoic acid (DPA)	Q1	3.4	547	149	Q1	1.00			Q1	1.00		
	Q2	7.9	548	125	Q2	0.79	0.60	1.04	Q2	0.80	0.58	1.09
	Q3	13.7	548	126	Q3	0.79	0.60	1.04	Q3	0.77	0.56	1.06
	Q4	28.0	547	87	Q4	0.51	0.38	0.68	Q4	0.54	0.38	0.78
*p*-value for trend						<0.0001				0.0012		
Docosahexaenoic acid (DHA)	Q1	42.6	547	146	Q1	1.00			Q1	1.00		
	Q2	92.3	548	123	Q2	0.79	0.60	1.05	Q2	0.83	0.60	1.13
	Q3	156.0	548	125	Q3	0.81	0.61	1.06	Q3	0.79	0.57	1.09
	Q4	306.4	547	93	Q4	0.57	0.42	0.76	Q4	0.61	0.42	0.88
*p*-value for trend						0.0003				0.011		

Model 1 adjusted for age, Model 2 adjusted for age, BMI, education level, household income, marital status, job, current alcohol drinking, current smoking, physical activity, chronic diseases status, sleep duration, family history of depression, stress, menopause status. and total energy intake. CI: confidence interval, Q: quartile.

**Table 4:** The correlation between the regions related to depression and omega-3 fatty acid intake among the networks.

Target		Omega-3 Fatty Acid	α-Linolenic Acid	Eicosapentaenoic Acid	Docosapentaenoic Acid	Docosahexaenoic Acid
Default mode network						
Right inferior parietal	*r*	−0.16	−0.06	−0.23	−0.22	−0.22
	*p*	0.165	0.641	0.053	0.064	0.055
Right olfactory	*r*	−0.19	−0.13	−0.19	−0.16	−0.19
	*p*	0.112	0.262	0.104	0.173	0.107
Right middle frontal	*r*	−0.32	−0.19	−0.35	−0.35	−0.35
	*p*	0.005	0.099	0.002	0.002	0.002
Left temporal superior pole	*r*	−0.13	−0.13	−0.10	−0.10	−0.08
	*p*	0.280	0.283	0.400	0.412	0.500
Dorsal attention network						
Right inferior temporal	*r*	−0.22	−0.10	−0.26	−0.30	−0.28
	*p*	0.060	0.386	0.026	0.009	0.017
Left inferior orbital frontal	*r*	−0.04	−0.04	−0.03	−0.05	−0.02
	*p*	0.741	0.751	0.806	0.691	0.856
Left middle cingulum	*r*	−0.28	−0.31	−0.15	−0.19	−0.15
	*p*	0.016	0.006	0.198	0.107	0.189
Right precuneus	*r*	−0.20	−0.12	−0.21	−0.22	−0.21
	*p*	0.094	0.291	0.077	0.059	0.075
Left middle occipital	*r*	−0.18	−0.16	−0.16	−0.12	−0.15
	*p*	0.118	0.173	0.168	0.305	0.198
Frontoparietal network						
Right middle temporal pole	*r*	−0.27	−0.10	−0.36	−0.38	−0.36
	*p*	0.019	0.389	0.002	0.001	0.001
Right middle occipital	*r*	−0.20	−0.14	−0.19	−0.19	−0.19
	*p*	0.092	0.226	0.102	0.100	0.103
Left cerebellar cortical crus II	*r*	−0.20	−0.18	−0.16	−0.12	−0.16
	*p*	0.094	0.125	0.184	0.327	0.176
